# Comparative Proteomics of the *Acanthopagrus schlegelii* Gonad in Different Sex Reversal

**DOI:** 10.3390/genes13020253

**Published:** 2022-01-28

**Authors:** Shuyin Chen, Yunxia Yang, Bo Gao, Chaofeng Jia, Fei Zhu, Qian Meng, Zhiwei Zhang, Zhiyong Zhang, Shixia Xu

**Affiliations:** 1 Marine Fisheries Research Institute of Jiangsu Province, Nantong 226007, China; shuyinchen89@163.com (S.C.); 22791326@163.com (B.G.); chaofeng.124@163.com (C.J.); ebancool@126.com (F.Z.); mq1992917@163.com (Q.M.); zhzhwei2005@126.com (Z.Z.); 2 Department of Aquaculture, Zhejiang Ocean University, Zhoushan 316022, China; yangyx@zjou.edu.cn; 3 College of Life Sciences, Nanjing Normal University, Nanjing 210023, China

**Keywords:** sex reversal, proteomics analysis, cathepsins, apoptosis

## Abstract

A substantial proportion of *Acanthopagrus schlegelii* individuals change sex from male to female during their lifetime. However, the mechanisms underlying sex change are unknown. In this research, iTRAQ analyses of proteins obtained from *A.* *schlegelii* gonads in four different stages of development were compared. In total, 4692 proteins were identified, including common sex-specific proteins, such as sperm-associated antigen 6 and cilia- and flagella-associated proteins in males, and zona pellucida sperm-binding proteins in females. Furthermore, proteins involved in the integrin signaling pathway, inflammation mediated by the chemokine and cytokine signaling pathways, pyruvate metabolism, CCKR signaling map, de novo purine biosynthesis and the ubiquitin proteasome pathway were upregulated in female gonads, whereas proteins implicated in DNA replication, the heterotrimeric G-protein signaling pathway, Gi alpha- and Gs alpha-mediated pathways, wnt signaling pathway, and hedgehog signaling pathway were upregulated in male gonads. Interestingly, cathepsins were only identified in ovaries, indicating their potential involvement in rapid ovarian development. Apoptosis-related proteins expressed in ovaries (such as MAPK and Cdc42) may protect them from cancer. This is the first report on the gonad proteome from *A.* *schlegelii* in different stages of sex reversal, and the results provide important fundamental data for studying the molecular mechanisms of sex reversal.

## 1. Introduction

The evolution of sex determination is an important scientific issue in evolutionary biology. Especially in fish, sex reversal is a common phenomenon and has aroused widespread interest among researchers. Fish are the only vertebrate group with functional hermaphroditism, where a substantial proportion of individuals in a population function as both sexes at some time during their life [1]. There are three types of hermaphroditism: protogyny, in which some or all individuals function first as females and later in life function exclusively as males; protandry, in which the sex change is from male to female; and simultaneous hermaphroditism, in which individuals function at the same time of life as both male and female [2]. In the protogyny and protandry types, sex reversal happens when an individual transforms their sex from male to female or female to male. Shao et al. [3] showed that epigenetic regulation plays multiple, crucial roles in the sex reversal of half-smooth tongue sole (*Cynoglossus semilaevis*). Since half-smooth tongue sole has both a genetic sex determination and an environmental sex determination, they suggest a causal link between the bias sex chromosome and the transgenerational epigenetic inheritance of sex reversal in tongue sole fish. In zebrafish (*Danio rerio*) and swamp eel (*Monopterus albus*), these species undergo natural sex changes from female to male via extensive morphological and physiological changes in the gonads [4,5,6]. Gonadal transformation is an ideal model system for sexual development in vertebrates. However, the molecular mechanism underling sex change in fish remains unclear.

The black porgy, *A. schlegelii*, is one kind of marine protandrous hermaphrodite fish with a multiple spawning pattern. The individuals are functional males for the first 2 years of life but begin to sexually reverse to females during their third year [7,8,9,10,11]. However, only some black porgies change to females, while the rest remain functional males during the third spawning season. Bisexual gonads with testicular and ovarian tissue separated by connective tissues were found in black porgies before they underwent sex change. The changes occurring in the gonad tissue are from a ‘bisexual gonad’ to ‘full ovarian gonad’ with primary oocytes and regressed testicular tissue, and then finally to an ‘advanced (vitellogenic) ovary’ [12]. Vitellogenic oocytes did not appear in the ovary until the complete regression of testicular tissue. This sex pattern provides a very good model to study the mechanism of sex change in fish. However, the molecular mechanism underling sex change in the black porgy remains poorly explored.

Recent research about sex reversal has identified a number of candidate sex-determining genes in various fish [13,14,15,16], such as *sf-1*, *dmrt1*, *dax-1*, *amh*, *wnt4*, *foxl2* and *cyp19a1a*. These genes were associated with testicular and ovarian development [17]. Additionally, estradiol, aromatase and steroid receptors in the gonadal tissue play important roles in the natural and controlled sex changes in black porgy [2,18]. Zhang et al. used a draft genome to analyze the sex changes in protandrous Chinese black porgy and demonstrated that three types of genes may be related to sex change: sex determination and differentiation, pluripotency factors, and apoptosis factors [19,20].

In the present study, protein profiles of black porgy gonads in the male, male-dominated hermaphroditism, female-dominated hermaphroditism and female phases were obtained. The compared results from each group were used to explore which proteins play the key role in the gonadal development, especially to examine the protein mechanism of sex reversal in the black porgy. The understanding of fish sex reversal mechanisms helps us to understand the evolution and development of fish reproductive strategies and the process of vertebrate sex determination.

## 2. Materials and Methods

### 2.1. Experimental Fish

The experimental fish were acclimated to pond conditions in seawater with a natural light system. The water temperatures ranged from 19 to 26 °C. The fish were fed with commercial feed. All procedures and investigations used in this study were conducted in accordance with regulations of China on the administration of laboratory animals. No specific permission was required for the species.

**Figure 1:**
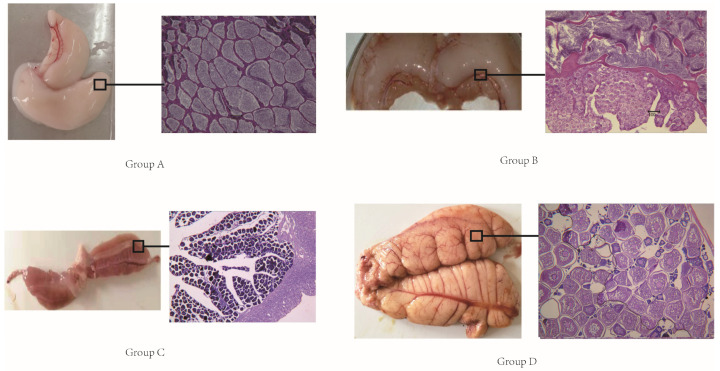
A picture of different sex phases and slices. Group (**A**): male gonads. Group (**B**): male-dominated hermaphroditic gonads. Group (**C**): female-dominated hermaphroditic gonads. Group (**D**): female gonads.

We collected gonads from black porgy in four different developmental stages and performed a histological slide. Combining extrinsic features with the histological slide, we determined them as: male gonads (group A), male-dominated hermaphroditic gonads (group B), female-dominated hermaphroditic gonads (group C), and female gonads (group D) (Figure 1). Gonads from group A fish appeared to be mature testes containing sperm. Gonads from fish in both groups B and C were hermaphroditic. Histological examination allowed the separation of these two groups such that male-dominated hermaphroditic gonads with matured sperm and early-stage ovarian germ cells were classified as functional males (group B), and gonads showing testicular degeneration and pronounced ovarian development were classified as functional females (group C). Gonads from fish in group D presented as mature ovaries with vitellogenic-stage oocytes.

### 2.2. Protein Extraction and Enzyme Hydrolysis

Gonad tissues from four batch groups, with three repeat samples in each group, were respectively homogenized to powder in liquid nitrogen. The powder was resuspended in a lysis buffer (7 M urea, 2 M thiourea, 4% SDS, 40 mM Tris-HCl), with a final concentration of 1 mM PMSF and 2 mM EDTA. After mixing, the samples were added to dithiothreitol (DTT) to a final concentration 10 mM, then sonicated at 200 W for 15 min. The supernatant was obtained following centrifugation at 13,000× *g* for 20 min at 4 °C and was added to 4 fold volume of ice-cold acetone and precipitated at −20 °C overnight. After centrifugation as described above, the pellets were dissolved in 400 mL, 100 mM TEAB and 7 M urea. The supernatant was reduced with 10 mM DTT and a water bath for 30 min at 56 °C. Next, the reaction was blocked with 55 mM IAM at dark room temperature for an alkylation reaction lasting 30 min. The homogenates were centrifuged, and the resulting supernatant protein samples were stored at −80 °C. The protein concentration was quantified by the Bradford method. A total of 100 μg protein from each sample was used for trypsin digestion. Briefly, trypsin was added to protein solution, which was diluted five times with 100 mM TEAB at 1:50 and hydrolyzed overnight at 37 °C. The peptides hydrolyzed by the enzyme were desalted by C18 column and then desalinated by vacuum freeze-drying.

### 2.3. iTRAQ Labeling and Grouping

Samples were dissolved with 0.5 M triethylammonium bicarbonate. According to the specification of the iTRAQ-8 marker kit, the samples were marked and mixed. The proteins from each sample were labeled with iTRAQ reagents 113, 114, 115 and 116, respectively. The labeled samples were pooled and purified using a strong cation exchange chromatography column (Phenomenex, Torrance, CA, USA) and separated by liquid chromatography (LC) using the Ultimate 3000 HPLC system, USA). Additionally, the chromatographic column used was Durashell C18 (5 μm, 4.6 × 250 mm). The peptide was separated by increasing an ACN concentration under alkaline conditions, and the flow rate was 1 mL/min. A total of 42 secondary components were collected and combined into 12 components. The combined components were desalted on the Strata-X column and dried under vacuum. A triple TOF 5600 liquid mass spectrometry system and liquid chromatography-mass spectrometry (LC-MS) were used for mass spectrometry data acquisition. Briefly, the polypeptide sample was dissolved in 2% acetonitrile/0.1% formic acid and analyzed by a Triple TOF 5600 plus mass spectrometer coupled with an Eksigent nano LC system. The polypeptide solution was added to the C18 capture column (5 μm, 100 μm × 20 mm) and eluted at a 90 min time gradient and at a flow rate of 300 nL/min on the C18 column (3 μm, 75 μm × 150 mm). For IDA (information-dependent collection), the primary mass spectrometry of 30 precursor ions was scanned with an ion accumulation time of 250 ms and the secondary mass spectrometry of 30 precursor ions was collected with the ion accumulation time of 50 ms. The MS1 spectra were collected in the range of 350 ≤ 1500 m.z., and the MS2 spectra were collected in the range of 100 ≤ 1500 m.z. The dynamic exclusion time of precursor ions was set to 15 s.

In this experiment, peptides were identified using protein-pilot TM V4.5, a search engine matching with AB Sciex 5600 plus, with the following paragon parameters. For the identification results of protein-pilot, we conducted further filtration using unused scores. We chose the peptides with unused scores ≥ 1.3, which means the reliability level is more than 95%. The filtrated proteins were used in the subsequent analysis.

### 2.4. Bioinformatic Analysis

Bioinformatic analysis was carried out to categorize proteins based on biological processes, cellular components and molecular function using annotations in the protein analysis through evolutionary relationships database v 6.1 (www.pantherdb.org, accessed on 1 April 2021) [21], which was in compliance with gene ontology (GO) standards. GO enrichment analysis showed the rich GO terms of differential proteins at the background of identified proteins, which connected the differential proteins to GO databases and computed the protein number of each corresponding GO terms. Next, the GO enrichment terms of differential proteins at the background of identified proteins were discovered by means of a hypergeometric test (*p* < 0.05). Pathway enrichment analysis of significant proteins was carried out using the Kyoto encyclopedia of genes and genomes (KEGG) database (http://www.genome.jp/kegg/, accessed on release 98.0, 1 April 2021). The pathway of significant enrichment was compared with all proteins that underwent hypergeometric testing; *p* < 0.05 and FDR < 0.05 were used as a threshold to select significant KEGG pathways.

## 3. Results

### 3.1. Protein Profiling and iTRAQ Quantification

**Figure 2:**
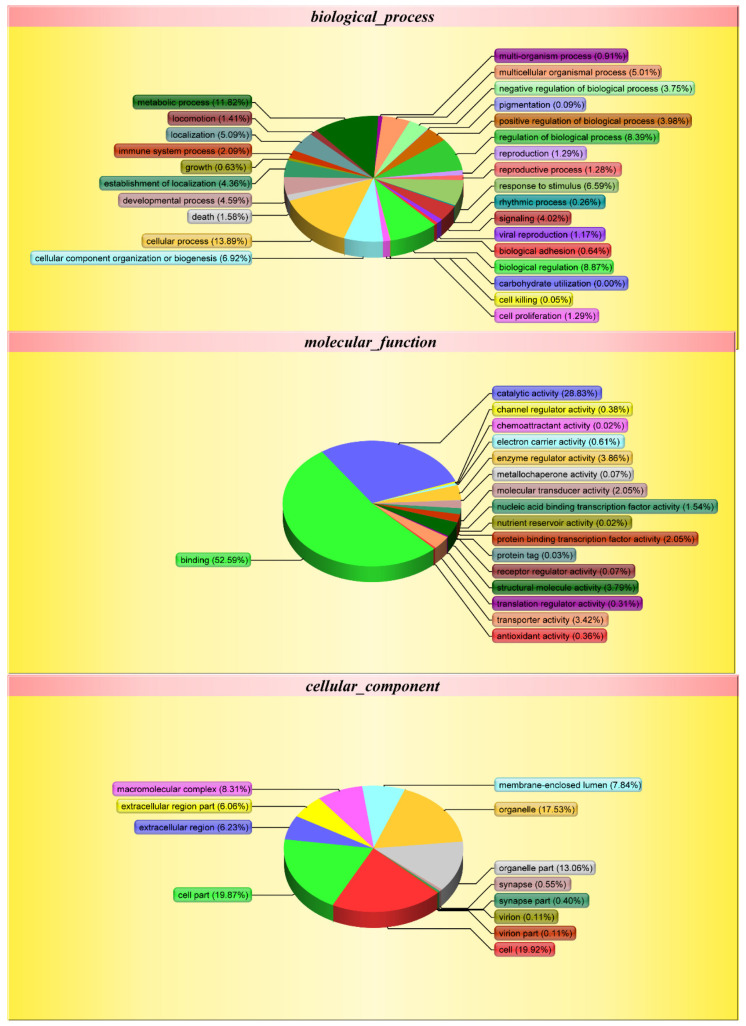
Gene ontology (GO) analysis of proteins. In total, 4692 proteins were identified by GO analysis. Shown above is the classification of these proteins into different categories based on biological process, molecular function and cellular component.

All MS/MS spectra were processed using Protein-pilot TM V4.5. As shown in Table 1, iTRAQ analysis of the black porgy proteome showed an average of 159,506 queries (163,323, 167,175 and 148,021 in batch 1, 2 and 3, respectively) in 3_frame_black_porgy_gene_proteins.fasta (58395 sequences), resulting in 4692 protein hits, which can be categorized with GO into diverse classes related to binding (52.59%), catalytic activity (28.83%), cell parts (19.87%), cellular processes (13.89%), metabolic processes (11.82%), biological regulation (8.87%) and macromolecular complexes (32.80%) (Figure 2).

The differential expression of the proteins was further quantified by iTRAQ analysis (Table 2). In total, 1559 proteins were further quantified in group A versus D, including 820 upregulated proteins and 739 downregulated proteins; 1354 proteins were further quantified in group A versus C, including 735 upregulated proteins and 619 downregulated proteins; and 1270 proteins were further quantified in group B versus D, including 748 upregulated proteins and 522 downregulated proteins. There were 675 up- and 538 down-regulated proteins in group B versus C, 481 up- and 622 down-regulated proteins in group C versus D and 70 up- and 80 down-regulated proteins in group A versus B.

### 3.2. Differentially Expressed Proteins with Panther Analysis

According to the differentially expressed protein results (Table 2), there were 820 up-regulated proteins in the male group (group A) that showed male features compared with group D, which showed female features. The PANTHER analysis showed that there were 18 protein classes: cytoskeletal protein (16.7%), metabolite interconversion enzyme (16.7%), nucleic acid-binding protein (16.0%), protein modifying enzyme (12.3%), transporter (6.2%), etc. These proteins were significantly enriched in 45 pathways, including Huntington’s disease (9.2%), ubiquitin proteasome pathway (7.9%), DNA replication (5.3%), heterotrimeric G-protein signaling pathway, Gi alpha and Gs alpha-mediated pathway (3.9%), wnt signaling pathway (3.9%), etc. There were seven significant up-regulated proteins: pachytene checkpoint protein 2 homolog (TRIP13), DNA topoisomerase 2-alpha (TOP2A), mitotic checkpoint BUB3, centromere protein X (CENPX), meiosis-specific coiled-coil domain-containing protein (MEIOC), fanconi anemia group D2 protein (FANCD2) and six opposite-strand transcript 1 (SIX6OS1), which appeared in the reproductive process (GO:0022414).

There were 739 down-regulated proteins in group A vs. group D. The PANTHER analysis showed that there were 15 protein classes in group D: metabolite interconversion enzyme (20.5%), nucleic acid-binding protein (22.4%), cytoskeletal protein (11.2%), protein-modifying enzyme (9.9%), transporter (6.2%), etc. These proteins belong to 51 pathways. Most proteins belong to the integrin signaling pathway (13.3%), inflammation mediated by chemokine and cytokine signaling pathway (6.7%), Huntington’s disease (5.0%), FGF signaling pathway (4.2%), gonadotropin-releasing hormone receptor pathway (4.2%), etc. There are four proteins in the gonadotropin-releasing hormone receptor pathway: vinculin (VCL), mitogen-activated protein kinase 3 (MAPK3), cell division control protein 42 homolog (Cdc42) and Ras-related C3 botulinum toxin substrate 1 (Rac1). These proteins showed us that cell division was active in group D. Additionally, we detected the protein zona pellucida sperm-binding protein 3 (ZP3), which belongs to the class of reproductive processes.

There were 619 down-regulated proteins in group C when compared with group A. The PANTHER analysis showed that there were 14 protein classes in group C up-regulated proteins: metabolite interconversion enzyme (28.8%), nucleic acid binding protein (17.1%), translational protein (13.5%), protein-modifying enzyme (11.7%), transporter (7.2%), etc. These proteins belonged to 32 pathways. Most proteins belonged to the integrin signaling pathway (15.6%), methylmalonyl pathway (6.7%), inflammation mediated by chemokine and cytokine signaling pathway (6.7%), glycolysis (4.4%), Huntington’s disease (4.4%), pyruvate metabolism (4.4%), CCKR signaling map (4.4%), de novo purine biosynthesis (4.4%), etc. The results showed that estradiol 17-beta-dehydrogenase 1, found in the androgen/estrogen/progesterone biosynthesis protein class, had high up-regulation in group C. Additionally, ZP3 was found in the reproductive processes (GO:0022414) of group C’s up-regulated proteins.

Compared with the female group, we identified 748 up-regulated proteins in group B. The PANTHER analysis showed that there were 11 protein classes involved in group B’s up-regulated proteins: nucleic acid-binding protein (29.2%), cytoskeletal protein (22.9%), protein-modifying enzyme (16.7%), chromatin/chromatin-binding or -regulatory protein (8.3%), transporter (6.3%), etc. These proteins belonged to 18 pathways, including Huntington’s disease (12.5%), the wnt signaling pathway (8.3%) and the hedgehog signaling pathway (8.3%). In addition, sperm-associated antigen 6 (Spag6) protein was found in cytoskeletal protein [22] for its function of regulating fibroblast cell growth, morphology, migration and ciliogenesis. This protein also localizes to the tail of permeabilized sperm [23]. This protein hinted that sperm cells were present in group B.

From the PANTHER analysis results, the differentially expressed proteins in each group focus on p38 MAPK pathway, p53 signaling pathway, PI3K-Akt signaling pathway, Huntington’s disease, ubiquitin proteasome pathway, TGF-beta signaling pathway, wnt signaling pathway, hedgehog signaling pathway, integrin signaling pathway, apoptosis signaling pathway, etc. Most of them were related to a wide spectrum of cellular functions, such as proliferation, apoptosis, differentiation and migration.

## 4. Discussion

Protandrous black porgy has a striking life cycle, with hermaphroditism at the juvenile stage and a male-to-female sex change at 0–3 years of age [7,8,24]. In the early stage of reproduction, the individual gonads showed male-dominated hermaphroditism. In the reproductive period, some of the individuals participated in reproductive activities as males, and the others were female individuals. At the end of reproduction, the male gonads rapidly transformed into the ovary-dominated hermaphroditic form, after which the gonad growth continued to proliferate and the spermary degenerated into a remnant, until all of the individuals appeared to be female from the appearance of the gonads [11]. Therefore, the black porgy is an ideal model for sex reversal.

### 4.1. Rapid Ovarian Development Mediated by Cathepsins and Immune System Protection from Cancer

At the stage of sex reversal, the male gonad could rapidly transform into the ovary-dominated hermaphroditic form, after which the gonad growth continued to proliferate and the spermary degenerated into a remnant [25]. Our results showed that differentially expressed proteins in group C were significantly enriched in several key pathways, including the integrin signaling pathway, inflammation mediated by the chemokine and cytokine signaling pathways, CCKR signaling, etc. The integrin signaling pathway, which is the specific binding of the extracellular domains to extracellular matrix (ECM), in some cases, to counter-receptors on adjacent cells, supports cell adhesion and is crucial for embryonic development, tissue maintenance and repair, host defense and hemostasis. These signals determine cellular responses such as migration, survival, differentiation and motility. Inflammation mediated by the chemokine and cytokine signaling pathways and CCKR signaling was focused on the defense reactions of the fish.

Importantly, some special proteins are worthy of attention. Several cathepsin members, including B, D, E, L and S, were detected to have significantly higher expression levels in group C when compared with group A and B. The human cysteine cathepsin is comprised of 11 members, including cathepsins B, C, F, H, K, W, X, L, O, S and Z; they display elaborate action in the degradation of native collagen and in the components of ECM [26]. Cathepsins B, L and S are key mammalian proteases that play an important role in the immune response. Cathepsin D and E and aspartic protease interact with other important molecules and influence cell signaling to regulate apoptosis. Cathepsin D is an estrogen-induced lysosomal protease [27]. The regulation of cathepsin D mRNA by estrogens and antiestrogens has been studied [28] Lysosome-mediated apoptosis is associated with cathepsin D [29]. Cathepsin E was expressed in Chinese hamster ovary cells [30]. Moreover, cathepsin D, secreted by cancer cells, acts as a mitogen on both cancer and stromal cells and stimulates their invasive and metastatic properties [31].

These more highly expressed cathepsins may be related to rapid ovarian development. The changes occurring in gonad tissue during the process of natural sex change are from a ‘bisexual gonad’ to a ‘full ovarian gonad’ with primary oocytes and regressed testicular tissue and then, finally, to an ‘advanced (vitellogenic) ovary’ [12]. The ECM is the principal structure of normal tissue and collagen proteins are the main structural proteins of ECM. High-level cathepsins may disrupt ECM remodeling by degrading the collagen proteins. The cathepsins degraded the ECM of ovarian tissue to promote rapid cell proliferation. Previous research has shown that cathepsins play a critical role in tumor invasion and metastasis by degrading ECM [32]. These results suggest that the gonads of group C are in the oogenesis stage. In group D, laminin and collagen proteins, which maintain the structure of ECM, had higher expression. These results hint that the process of ovary formation was associated with ECM degradation and remodeling mediated by cathepsins. This process is similar to the tumor invasion and metastasis.

Interestingly, the proteins in inflammation mediated by the chemokine and cytokine signaling pathways were activated in group C. Perhaps to prevent the rapid dividing cells from becoming cancerous, fish evolved an efficient immune system to protect itself from cancer. Mitogen-activated protein kinase (MAPK) and cell division control protein 42 (Cdc42), which belong to inflammation mediated by the chemokine and cytokine signaling pathways, were detected in group C, compared with A and B. Current evidence clearly shows that MAPK pathways are viable targets for cancer therapy [33]. Additionally, some research has found that Cdc42 plays an essential role in regulating multiple cellular processes including cell proliferation, division, migration, morphogenesis, and especially epithelial polarity establishment [34]. The highly expressed proteins in this pathway potentially protect ovary tissues from cancer.

In male-dominated hermaphroditism, oocytes survive in the testes by altering the soma fate from male to female in the protandrous black porgy. HSD17B1 protein, which was detected in our research, has a dual function in estrogen activation and androgen inactivation and plays a major role in establishing the estrogen E2 concentration gradient between serum and peripheral tissues [35]. Additionally, the gene of this protein is expressed primarily in the placenta and ovarian granulosa cells [36]. Lee [37] showed that aromatase inhibitors block natural sex change and induce male function in the protandrous black porgy. The aromatase protein, which can convert androgens to estrogens, was detected in group C and D, but was not expressed significantly higher than in other groups.

### 4.2. Male-Specific and Female-Specific Proteins Separately Identified in Hermaphroditic Fish

Our proteomics results also showed that more proteins related to oogenesis, such as ZP3 and ZP4, were up-regulated in groups C and D. The zona pellucida is an extracellular matrix that surrounds the oocyte. The proteins of fish egg envelopes are encoded by genes that are closely related to the genes for human zona pellucida proteins [38]. ZP3 has only been detected in immature testes and ovaries [39,40]. ZP3 is an essential molecule for the binding of sperm to the egg envelope and for the induction of the acrosome reaction [39], and it is localized in the inner surface of the egg envelope [41]. Similar localization is only shown in fish, suggesting that ZPs are unique molecules which may allow us to investigate the functional evolution of the egg envelope in vertebrates [42,43].

During the reproductive season, the flagellum developed from the distal centriole of a cytoplasmic diplosome in spermatids [11] in groups A and B. The results showed that some proteins in groups A and B belonged to the wnt signaling pathway and the hedgehog signaling pathway. These two pathways were associated with male function [44,45]. Protein SIX6OS1, which showed significant up-regulation in group A, participates in not only the meiotic process, but also germ cell development and spermatogenesis. It is the central element of the synaptonemal complex and is essential for mouse fertility [46]. Additionally, we found that the dosage suppressor of mck1 homolog (Dmc1) [47] and synaptonemal complex protein (Sycp3), which are specifically expressed during early meiotic prophase I [48], are up-regulated proteins in group A. Spag6 protein also was found in group A and B. This protein also localizes to the tail of permeabilized sperm [22]. The Spag-deficient males surviving to maturity were infertile [49]. Significantly, the cilia- and flagella-associated protein family (CFAPs) genes had high up-regulated expression in groups B and C. CFAPs belong to the cell part, and their molecular function is binding. Diseases associated with CFAPs include spermatogenic failure and non-syndromic male infertility due to sperm motility disorder. CFAP43 and CFAP44 are specifically or preferentially expressed in the testes [50]. Additionally, some studies have shown that loss-of-function mutations in members of CAFPs can lead to male infertility in humans and mice [51]. We also detected the doublesex- and mab-3-related transcription factor 1 (Dmrt1) protein, which plays a key role in the testicular development and spermatogonia proliferation of black porgy [52]. It is more up-regulated in group A than in other groups, but not significantly. These results revealed that there are many proteins that are related to spermatogenesis.

Overall, the results of this study hint that the production of sex-specific marker proteins provides corresponding functions for organisms. In our research, cathepsins had high expression levels during sex reversal, which may be used to degrade the ECM. ECM degradation and remodeling may be associated with ovary formation. Zhang et al. demonstrated that three types of gene may be related to sex change: those for sex determination and differentiation, pluripotency factors, and apoptosis factors [19,20]. From our results, ECM degradation and remodeling is perhaps related to apoptosis. As well as eliminating cells that are detached from their ECM, this mechanism of apoptosis also plays an important role in development and tissue remodeling [53].

In general, proteomics can be used to identify proteins and compare the protein expression levels of different groups; it is the study of the entire complement of proteins expressed spatially and temporally in an organism. Wu et al. [54] demonstrated the molecular and cellular regulation of sex change in hermaphroditic fish, *A. schlegelii*. They showed that the sex change is regulated by the male fate. Furthermore, the hypothalamus (Gnrh)-pituitary (Gths) axis and testicular *dmrt1* play an important role in sex fate decision in black porgy. In *A. schlegelii*, removing testes in fish show a precocious femaleness with the alternation in the epigenetic modification of *cyp19a1a* promoter. The Dmrt was detected in groups A and B of our research. Furthermore, we found the aromatase in group C and D. Tsakogiannis et al. [55] obtained information from comparative transcriptomics in sparidae hermaphrodites. Their results showed that *zp3* and *zp4* were commonly over-expressed in the ovaries of all five sparidae hermaphroditic species, which is consistent with our results. Additionally, they found the testis-associated protein, Spag, as well as spermatogenesis-associated proteins, such as sperm flagellar-related protein, which were also found in our research.

## Figures and Tables

**Table 1:** Summary of protein identification information.

Group Name	Batch1	Batch2	Batch3
Total spectrum number	412,850	410,455	409,324
Identification spectrum number *	163,323	167,175	148,021
Spectral identification rate	39.79%	40.73%	36.16%
Identification of peptide number *	26,898	28,338	23,513
Identification of protein number	3967	4168	3736
Unique-2 **	3274	3432	3020

Note: * indicates that credibility is at least 95%, ** indicates the number of identified proteins with at least two unique peptides.

**Table 2:** The number of significantly different proteins between the two samples.

Compared Group	A/B	A/C	A/D	B/C	B/D	C/D
Up-regulated protein number	70	735	820	675	748	481
Down-regulated protein number	80	619	739	538	522	622
Total different protein number	150	1354	1559	1213	1270	1103

## Data Availability

The authors confirm that the data supporting the findings of this study are available within the article.
